# Bifurcation Type and Larger Low Shear Area Are Associated with Rupture Status of Very Small Intracranial Aneurysms

**DOI:** 10.3389/fneur.2016.00169

**Published:** 2016-11-24

**Authors:** Yisen Zhang, Zhongbin Tian, Linkai Jing, Ying Zhang, Jian Liu, Xinjian Yang

**Affiliations:** ^1^Department of Interventional Neuroradiology, Beijing Neurosurgical Institute and Beijing Tian Tan Hospital, Capital Medical University, Beijing, China

**Keywords:** very small intracranial aneurysm, hemodynamics, rupture, wall shear stress, aneurysm type, bifurcation type

## Abstract

**Background:**

Characterization of the risk factors for rupture of very small intracranial aneurysm (VSIA, ≤3 mm) is clinically valuable, since VSIAs are implicated in subarachnoid hemorrhage. The aim of this study was to identify morphological and hemodynamic parameters that independently characterize the rupture status of VSIAs.

**Methods:**

We conducted a retrospective study of consecutive VSIAs between September 2010 and February 2014 in our institute. A series of morphologic and hemodynamic parameters were evaluated using computational fluid dynamics, based on patient-specific three-dimensional geometrical models.

**Results:**

We identified 186 patients with 206 VSIAs (73 ruptured, 133 unruptured). Univariable logistic regression analysis showed that bifurcation type, parent artery diameter, size ratio, time-averaged wall shear stress (WSS), maximum WSS, minimum WSS, and low shear area (LSA) were related to rupture status. Bifurcation type and larger LSA were independently associated with rupture status in multivariable logistic regression (*p* = 0.002 and *p* = 0.003, respectively).

**Conclusion:**

Bifurcation type and larger LSA were independently associated with VSIA rupture status. Further studies are needed prospectively on patient-derived geometries prior to rupturing based on large multi-population data to confirm the present findings.

## Introduction

Very small intracranial aneurysms (VSIAs, ≤3 mm) represent ~13.2–15.1% of all intracranial aneurysms (1, 2). According to a report by the International Study of Unruptured Intracranial Aneurysms (3), the risk of rupture of small (<7 mm) anterior circulation aneurysms was 0.1%/year. However, it was not uncommon for VSIAs to be responsible for subarachnoid hemorrhage (2). With developments in imaging, diagnosis of asymptomatic VSIAs has increased. Ruptured aneurysms must be isolated from the cerebral circulation. However, treatment of unruptured VSIAs remains controversial. The decision to treat an unruptured VSIA must factor in the risk of treatment: both microsurgical clipping and endovascular coiling (4). Thus, characterizing the features of aneurysms associated with rupture status is clinically important.

Recent studies have demonstrated that morphological and hemodynamic factors, including aneurysm size, neck size, size ratio (SR), aspect ratio (AR), wall shear stress (WSS), and oscillatory shear index (OSI), are associated with the rupture status of intracranial aneurysms (5–8). However, no previous study has evaluated the morphological and hemodynamic characteristics associated with rupture status from a cohort of VSIAs. Additionally, hemodynamic results are conflicting as both low and high WSS were reported to be associated with rupture status. Evidence shows that combined samples, including different sizes of aneurysm, might influence hemodynamic results in the assessment of rupture status, with larger aneurysms usually being characterized with low WSS (9, 10). The confounding effect of size should be controlled in the analysis of rupture status of aneurysms.

The aim of the present study was to identify the morphological and hemodynamic parameters that independently characterize the rupture status of VSIAs, using patient-specific computational fluid dynamics (CFD).

## Materials and Methods

The Ethics Committee of Beijing Tian Tan Hospital approved this study. Written informed consent was obtained from each study patient.

### Patient Population

From September 2010 to February 2014, angiography images of patients with intracranial aneurysms that were diagnosed or treated and present in our database were carefully reviewed. The aneurysm size was defined as the maximum perpendicular height of an aneurysm. The largest aneurysm size was measured by 2D or 3D angiography, and only patients with aneurysm size ≤3 mm were included in the present study. This criterion was based on the adopted definition of very small or tiny aneurysms in most published studies (2, 4, 11). For patients with multiple aneurysms, clinical and radiological information (such as blood distribution on CT scan) was considered, and a judgment of the most likely source of hemorrhage made. Subsequently, 186 patients with 206 very small aneurysms were included. Of the 206 aneurysms, 73 were ruptured, and 133 were unruptured. All geometries were reconstructed from 3D digital subtraction angiography. For anterior communicating artery aneurysms, cases with contralateral hypoplastic A1 segment were reconstructed directly using 3D digital subtraction angiography images. Otherwise, the anterior communicating artery aneurysm geometries were reconstructed using fusing bilateral 3D images of the internal carotid artery, using the rigid registration method or from CT angiography. All angiographic data within the subarachnoid hemorrhage were obtained in the emergent fashion prior to vasospasm.

### Numerical Modeling

**Figure 1:**
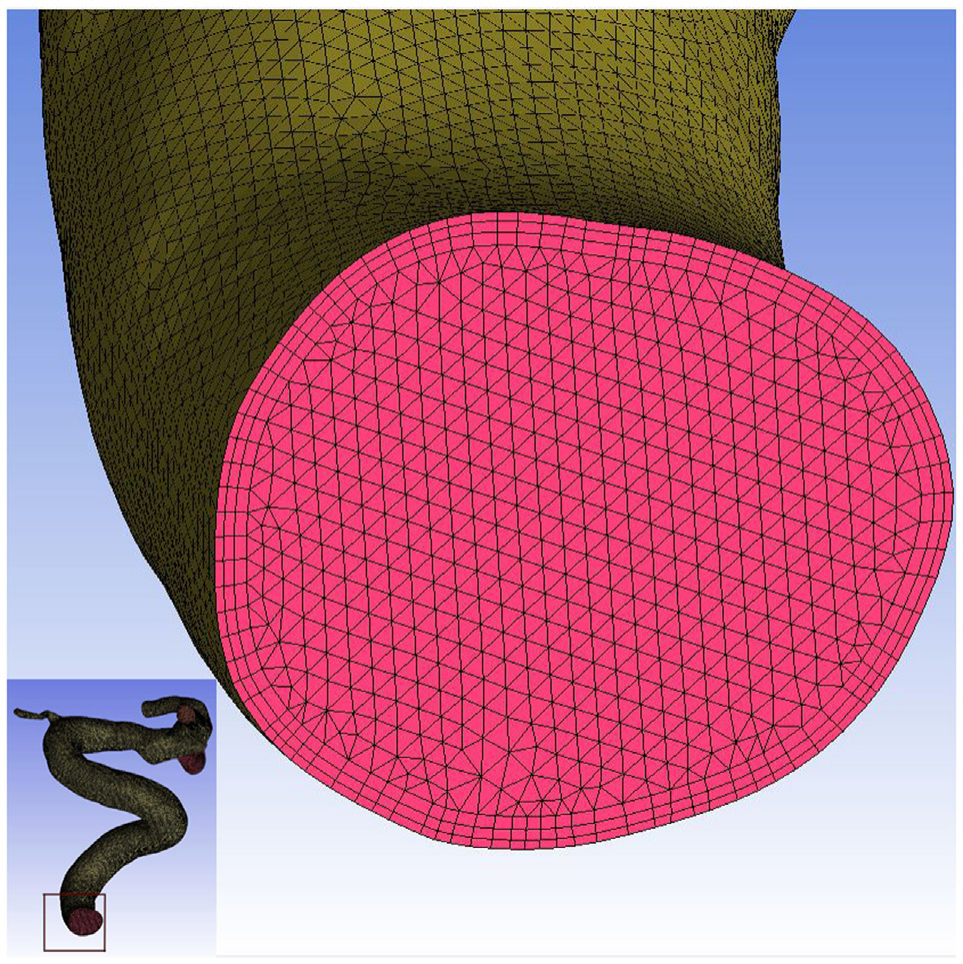
**Mesh generation in this study**. Three layers of prismatic grid were generated near the wall, and the tetrahedron grid was generated in the other field.

Each aneurysm model was imported into the automatic mesh generation software (ICEM CFD, ANSYS Inc., Canonsburg, PA, USA) to create more than 1 million finite volume element grids for CFD simulations. To ensure flow was accurately solved near walls, a hybrid grid was applied with three layers of prismatic grid near the wall, and a tetrahedron grid in the other field (Figure 1). Software, ANSYS CFX 14.0 (ANSYS Inc.), was then used to solve the flow governing Navier–Stokes equations with the assumption of laminar, incompressible, and Newtonian fluid. The density and dynamic viscosity of blood were specified as 1060 kg/m^3^ and 0.004 N s/m^2^, respectively. Blood vessel walls were assumed to be rigid with no-slip boundary conditions. According to vessel location, pulsatile velocity profiles obtained by transcranial Doppler from a normal patient were applied for inflow boundary conditions. Flow waveforms were scaled to achieve a mean inlet WSS of 15 dyne/cm under pulsatile conditions (12, 13). Traction-free boundary conditions were implemented at the outlet. Three cardiac cycle simulations were performed for numerical stability, and the last cardiac cycle was collected as output.

### Data Collection

Morphological parameters, including aneurysm size, neck size, aneurysm surface area, parent artery diameter, size ratio (SR, aneurysm dome-parent artery ratio), and AR (dome–neck ratio) were calculated and measured in 3D digital subtraction angiography images. The aneurysm type was recorded as sidewall or bifurcation (aneurysms originating from only one parent vessel or from the origin of a small branch whose caliber is less than one-fifth of the parent vessel are classified as lateral aneurysms; otherwise, bifurcation aneurysms) (5). Aneurysm dome shape was categorized as regular or irregular (irregular shape was recorded as aneurysm with irregularities due to bilobate bleb, polylobate bleb, or aneurysm wall protrusions) (14).

Hemodynamic parameters, including normalized time-averaged WSS (TAWSS), maximum WSS (maxWSS), minimum WSS (minWSS), OSI, and low shear area (LSA), were calculated based on simulated pulsatile flow simulations. In this study, LSA was calculated as the area of the aneurysm wall exposed to a TAWSS below 0.4 pa, and then normalized by aneurysm sac area (13).

### Statistical Analysis

Data are presented as mean and SD for quantitative parameters, and as frequency for categorical parameters. The Kolmogorov–Smirnov test for normal distribution was performed for all quantitative parameters. Student’s *t*-test was used if a parameter was normally distributed; otherwise, a Mann–Whitney *U* test was used to compare differences between ruptured and unruptured lesions. For categorical parameters, the chi-square test, and Fisher’s exact test, as appropriate, were used to analyze the data. Factors with *p* ≤ 0.2 in univariable analysis were entered into a multivariable logistic regression analysis. Results were considered statistically significant at *p* < 0.05. Statistical analysis was carried out using SPSS, Version 21.0 (SPSS, Chicago, IL, USA).

## Results

### Baseline Characteristics

Mean age of the patients was 53.0 ± 10.2 years (range, 22–79 years), and 64% (119/186) were women. Mean aneurysm size was 2.50 ± 0.46 mm (range, 1.3–3.0). A total of 35.4% (73/206) VSIAs were ruptured, and 62% (127/206) were sidewall aneurysms. The distribution of aneurysm location and aneurysm type is shown in Table 1.

**Table 1:** **Aneurysm locations and types (*N* = 206)**.

Location	No. of cases	Sidewall		Bifurcation	
		Ruptured	Unruptured	Ruptured	Unruptured
ICA	79	16	62	1	0
PcomA	47	7	26	7	7
AcomA	27	0	1	16	10
MCA	25	2	1	7	15
ACA	13	2	6	4	1
BA	8	0	0	4	4
PCA	3	3	0	0	0
PICA	4	1	0	3	0
Total	206	31	96	42	37
		127		79	

*ICA, internal carotid artery; PcomA, posterior communicating artery; AcomA, anterior communicating artery; MCA, middle cerebral artery; ACA, anterior cerebral artery; BA, basilar artery; PCA, posterior cerebral artery; PICA, posterior inferior cerebellar artery*.

### Differences in Morphologic Measurements between Ruptured and Unruptured VSIA Groups

As shown in Table 2, ruptured VSIAs had significantly larger SR (*p* = 0.001) and smaller parent artery diameter (*p* = 0.001) compared with unruptured lesions. Ruptured VSIAs were usually located at the bifurcation site (*p* < 0.001), and had irregular dome shape. However, aneurysm shape was not significantly different between the groups (*p* = 0.061). The remaining morphological parameters were not significantly different between ruptured and unruptured VSIAs.

**Table 2:** **Differences in morphologic and hemodynamic measurements between ruptured and unruptured VSIAs**.

Characteristics	Ruptured (73)	Unruptured (133)	Univariable *p*-value
Aneurysm size, mm	2.50 ± 0.47	2.51 ± 0.45	0.89
Neck size, mm	2.37 ± 0.60	2.46 ± 0.53	0.22
Parent artery diameter, mm	2.73 ± 0.96	3.19 ± 0.90	0.001
SR	1.04 ± 0.43	0.86 ± 0.31	0.001
AR	1.10 ± 0.30	1.06 ± 0.30	0.16
Aneurysm area, mm^2^	22.2 ± 10.7	23.4 ± 9.25	0.38
Irregular shape, *n* (%)	16 (21.92)	16 (12.03)	0.06
Bifurcation type, *n* (%)	42 (57.53)	37 (27.82)	<0.001
TAWSS, pa	3.26 ± 2.82	5.00 ± 3.80	<0.001
maxWSS, pa	11.6 ± 7.7	16.8 ± 10.4	<0.001
minWSS, pa	0.42 ± 0.71	0.55 ± 0.58	<0.001
OSI	0.012 ± 0.01	0.01 ± 0.01	0.28
LSA, %	19.8 ± 28.2	6.19 ± 16.2	<0.001

*SR, size ratio; AR, aspect ratio; TAWSS, time-averaged wall shear stress; maxWSS, maximum wall shear stress; minWSS, minimum wall shear stress; OSI, oscillatory shear index; LSA, low shear area; OR, odds ratio; CI, confidential interval*.

### Differences in Hemodynamic Measurements between Ruptured and Unruptured Groups

**Figure 2:**
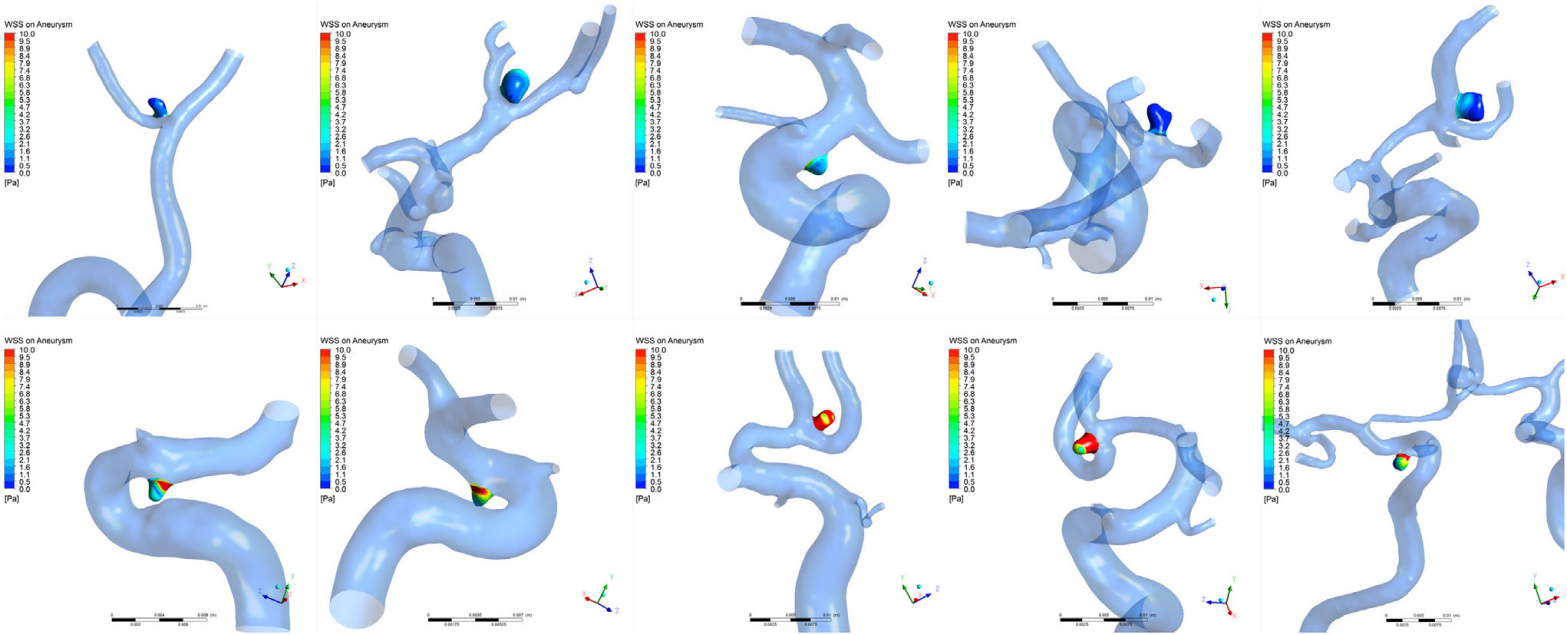
**Time-averaged wall shear stress distribution on very small intracranial aneurysms**. The top row shows a series of ruptured aneurysms and the bottom row shows a series of unruptured aneurysms. Wall shear stress was significantly lower in ruptured very small intracranial aneurysms compared with unruptured aneurysms.

The distributions of WSS for ruptured and unruptured VSIAs are shown in Figure 2. As demonstrated in Table 2, all hemodynamic parameters were significantly different between the groups, except OSI (*p* = 0.28). Ruptured VSIAs had significantly lower TAWSS (*p* < 0.001), maxWSS (*p* < 0.001), minWSS (*p* < 0.001), and higher LSA (*p* < 0.001) compared with unruptured VSIAs.

### Multivariable Logistic Regression Analysis of Risk Factors for VSIA Rupture

Multivariable logistic regression analysis was performed to identify independent parameters significantly correlated with VSIA rupture status (Table 3). Aneurysm type (sidewall/bifurcation) and LSA were retained as independently significant parameters associated with rupture status (*p* = 0.002, OR: 0.36, 95% CI: 0.19–0.68; *p* = 0.003, OR: 0.98, 95% CI: 0.96–0.99, respectively).

**Table 3:** **Multivariable logistic regression analysis of risk factors for rupture of VSIAs**.

Characteristics	Multivariable *p*-value	OR	95% CI
Parent artery diameter, mm	0.72	1.12	0.61–2.05
SR	0.86	0.87	0.18–4.13
AR	0.64	0.75	0.22–2.53
Irregular shape, *n* (%)	0.20	1.78	0.73–4.35
Bifurcation type, *n* (%)	0.002	0.36	0.19–0.68
TAWSS, pa	0.79	1.03	0.81–1.32
maxWSS, pa	0.13	1.07	0.98–1.16
minWSS, pa	0.06	0.46	0.21–0.98
LSA, %	0.003	0.98	0.96–0.99

*SR, size ratio; AR, aspect ratio; TAWSS, time-averaged wall shear stress; maxWSS, maximum wall shear stress; minWSS, minimum wall shear stress; LSA, low shear area; OR, odds ratio; CI, confidential interval*.

## Discussion

In this study, we showed that aneurysm type and LSA were significantly different between ruptured and unruptured VSIAs. Moreover, ruptured VSIAs were more likely to be located at bifurcation sites, with markedly low WSS. This finding may be clinically important for the evaluation of risk of VSIA rupture. In VSIAs that are equally suitable for observation or endovascular treatment, being located at bifurcation with markedly low WSS may guide management and more prompt intervention.

The mechanisms of intracranial aneurysm rupture have been studied with regard to morphologic and hemodynamic characteristics (8, 15–18), but aneurysms with different size, rather than relative uniform size, were investigated in these studies. This might explain previous conflicting findings. In an attempt to eliminate potential bias from size differences, we focused only on VSIAs in the present study.

A previous study reported low rupture risk for small aneurysms (3). However, VSIAs are often implicated in subarachnoid hemorrhage (1, 2). Treatment decisions for unruptured lesions are complicated because of the high risk of intra-operative complications (4). Therefore, clarification of the characteristics associated with rupture status of VSIAs is clinically important. In the current study, we found that ruptured VSIAs were more likely to be located at bifurcation sites with markedly low WSS. This finding may provide a simple method to predict bleeding risk in patients with unruptured VSIAs, and provide tailored treatment for such patients.

Many studies have demonstrated that bifurcation aneurysms are associated with aneurysm formation (19, 20), but few studies focus on correlations between aneurysm type (bifurcation or sidewall) and rupture. Baharoglu et al. (5) found that there was a dichotomy between sidewall and bifurcation aneurysms in identifying rupture status of intracranial aneurysms. Aneurysm type was included as an important morphologic parameter and evaluated in the current study, and our results showed that bifurcation type was independently associated with rupture status of VSIAs. A similar result was reported in Baharoglu et al. (5). The authors used CFD analysis in two generic aneurysm models (sidewall and bifurcation type), and found a fundamental difference in hemodynamics between sidewall and bifurcation aneurysms, which might be the reason why bifurcation type was more likely to rupture.

Rupture status of intracranial aneurysms can be successfully characterized by hemodynamic factors. However, results have been controversial for two theories explaining the mechanisms of intracranial aneurysm rupture, namely, high and low WSS. One reason for controversy might be that most studies simulated a full-range of aneurysm size in the sample studied. For example, it was reported that aneurysm size was a confounding factor in WSS rupture discrimination (2, 9). Thus, some studies eliminated the confounding effect of aneurysm size using size-match analysis. However, few such investigations have been published. To the best of our knowledge, the present study is the first and largest report of hemodynamic factors associated with rupture status of VSIAs.

This study also showed that LSA was independently associated with VSIA rupture status, which supports the low WSS theory. These data are consistent with our previous results showing that larger LSA might be associated with increasing risk of rupture (21). Pereira et al. (7) performed a case–control study in small aneurysms (mean, 3.7 mm) prone to rupture. Although no significant differences were found, all WSS-related parameters were lower in aneurysms that were prone to rupture. Ruptured aneurysms more frequently have larger LSA on the dome. Previous studies found that the thin-walled dome regions of unruptured aneurysms co-localized with low WSS, and bleeding points of ruptured aneurysms were often located at the dome with low WSS (22). Low WSS can cause dysfunction of flow-induced NO, increase endothelial permeability, and trigger atherosclerotic and inflammatory pathways (8, 13). Subsequently, degradation of the aneurysm wall occurs and ultimately leads to rupture. Hemodynamic factors may help us to identify ruptured aneurysms and offer insight into rupture mechanisms. Although results from the present study support the low WSS theory, the controversial result of WSS in stratifying the rupture status of intracranial aneurysms remains. The high WSS theory purports that high WSS initiates a different pathway to wall degradation, and should also be explored for association with rupture (8, 23).

The present study has some limitations that must be considered. First, although we focused on a subset of aneurysms (VSIAs) that removed the confounding effect of aneurysm size, all cases came from a single center and the number of cases was still smaller than optimal. Therefore, the findings may only be applicable to the specific size category studied, and may not be applicable to larger aneurysms. Second, this was a retrospective study, and the flow characteristics of pre-ruptured aneurysms may be different to post-ruptured aneurysms. Moreover, aneurysm geometry may have been affected by the rupture event. Such a theoretical possibility may have caused bias in the present results. A large-scale prospective randomized study should be performed to validate the morphological and hemodynamic findings in unruptured VSIAs that eventually rupture. Third, the mechanisms of aneurysm rupture cannot be explained wholly by hemodynamics or morphology. Other factors, such as genetic factors and clinical history, may also play an important role in the development of aneurysms. Fourth, similar to previous CFD studies (8), several assumptions, including rigid wall, laminar flow, Newtonian fluid, patient-specific inlet flow velocity, and traction-free outlet boundary conditions were made in our simulation, which may affect the generalizability of the hemodynamic results.

## Conclusion

In the largest population of VSIAs studied to date, bifurcation type and larger LSA were independently associated with VSIA rupture status. Further studies are needed prospectively on patient-derived geometries prior to rupturing or leaking based on large multi-population data to confirm the utility of the present findings.

## Author Contributions

YsZ carried out the simulation study and drafted the manuscript. JL, ZT, and YZ performed data collection and data analysis. XY and JL participated in the design of this study and helped to check the manuscript.

## Conflict of Interest Statement

The authors declare that the research was conducted in the absence of any commercial or financial relationships that could be construed as a potential conflict of interest.

## Abbreviations

AR, aspect ratio (dome-neck ratio); CFD, computational fluid dynamics; LSA, low shear area; OSI, oscillatory shear index; SR, size ratio (aneurysm dome-parent artery ratio); TAWSS, time-averaged wall shear stress; VSIA, very small intracranial aneurysm; WSS, wall shear stress.
